# Editorial: Research on low dose ionizing radiation health effects

**DOI:** 10.3389/fpubh.2025.1566179

**Published:** 2025-02-20

**Authors:** Jelena Pajic, Mirta Milic, Vladimir Jurisic, Volodymyr Vinnikov

**Affiliations:** ^1^Serbian Institute of Occupational Health, Belgrade, Serbia; ^2^Institute for Medical Research and Occupational Health, Zagreb, Croatia; ^3^Faculty of Medical Sciences, University of Kragujevac, Kragujevac, Serbia; ^4^Cancer Research Institute, Biomedical Research Centre of Slovak Academy of Science, Bratislava, Slovakia

**Keywords:** ionizing radiation, health effects, low doses, dose rate, biological response

Research Topic “*Research on Low Dose Ionizing Radiation Health Effects”* aimed to summarize current achievements in the field of low-dose health effects and biological response research, clarify the role of different approaches, and give an outline and directives for future projects.

## Introduction

Biological effects of low doses of ionizing radiation have remained a radiobiological enigma for decades. Researchers' opinions in this field range from “supralinear dose dependence” and “extreme hazard” to “adaptive response” and “radiation hormesis.” Here we'd like to present a new set of reports on the experimentally evaluated impact of low radiation doses.

## Highlights from the Research Topic

The first contribution examined short- and long-term effects of radiation exposure at low dose and low dose rate in normal human VH10 fibroblasts. In this study, human VH10 fibroblasts were exposed to three low doses of gamma radiation at four different dose rates in order to exmine gene expression and cytotoxic effects. Cell viability assays, performed 1, 3, and 6 days post irradiation, and colony forming assay, seeded just after exposure, did not reveal any statistically significant early effects on cell growth or survival patterns. Tendencies of increased viability (day 6) and reduced colony size (day 21) were observed at higher dose rates. Furthermore, no long-term changes were observed in cell growth curves generated up to 70 days after exposure. In conclusion, low doses of gamma radiation given at low dose rates had no strong cytotoxic effects on radioresistant VH10 cells (Akuwudike et al.; Frontiers Production Office).

In the second research rats were exposed gamma radiation at a low dose rate (LDR) of 6 mGy/h and a high dose rate (HDR) of 20 mGy/h for 30 days (5 h/day). Functional imaging was performed to assess the brain inflammation and blood–brain barrier (BBB) destruction of rats. Histological and immunofluorescence analyses were used to reveal the neuron damage and the activation of microglia and astrocytes in the hippocampus. RNA sequencing was conducted to investigate changes in gene expression in hippocampus. Following all performed analytical methods, it was concluded that compared with chronic low-dose γ-irradiation at HDR, LDR induced more severe cognitive impairment which might involve PI3K/Akt signaling pathway (Ma et al.).

The third publication examined biological effects in normal human fibroblasts following chronic and acute irradiation with both low- and high-LET radiation and concluded that alpha particles were more effective than gamma rays at inducing cytogenetic damage and reduced clonogenic cell survival. For gamma rays, both effects were greater in acute irradiation scenario, while for alpha particles equal cytogenetic damage and reduction of clonogenic cell survival for both chronic and acute exposure was found, except for the highest doses, where cytogenetic damage is highest at low dose rate. The results of this study may have an impact on space and terrestrial radioprotection of humans at low doses and low dose rates, on biodosimetry, and on the use of ionizing radiation in medicine. These results also provide insights into understanding damage induction and cell reaction mechanisms following chronic exposure (at dose rates as low as 18 and 5 mGy/h) to low- and high-LET radiation (Anello and Esposito).

## Conclusion

Ionizing radiation has been widely used in medical (radiotherapy and diagnostics) and occupational settings, where the effects of exposure to low doses play an important role in consideration of the health effects and biological response to radiation. The health risks of low doses in humans may not be accurately estimated by any current mathematical model because of numerous inherent, environmental, dietary, and biological variables that cannot be accounted for in epidemiologic studies. In addition, the expression of radiation–induced damage depends not only on dose, dose rate, linear energy transfer, fractionation, and protraction but also on repair mechanisms, bystander effects, and exposure to chemical and biological mutagens, carcinogens, tumor promoters, toxins, radioprotective substances. Therefore, low doses may not be considered insignificant risks for somatic and heritable mutations and disease (neoplastic and nonneoplastic) in humans. According to a well-established radiobiological concept, no radiation doses can be considered completely safe and all efforts must be made to reduce both the radiation dose and damage, no matter how small.

